# Influence of litter substrates on production, egg quality, and welfare indicators in laying hens

**DOI:** 10.1016/j.psj.2025.106201

**Published:** 2025-12-05

**Authors:** Nathaniel Nutsugah, Emma Ivarsson, Anette Wichman, Helena Wall

**Affiliations:** Department of Applied Animal Science and Welfare, Swedish University of Agricultural Sciences, Box 7024, SE-750 07 Uppsala, Sweden

**Keywords:** Litter substrate, Laying hen, Biochar, Peat, Animal welfare

## Abstract

Hens’ welfare indicators such as integument condition, dustbathing, and foraging behaviors can be influenced by the litter substrate, however, the resultant impact of litter substrate on performance and egg production remains unclear. Hence, this study investigated the impact of 4 litter substrates on various welfare parameters and productivity in laying hens. These substrates included wood shavings, peatmix (wood shavings mixed with peat), biochar (wood shavings amended with biochar), and microbial additive (wood shavings amended with microbial additive). A total of 1600 Bovan White hens were housed in groups of 100 in 16 identical pens (floor housing system). Treatments were randomly assigned to the pens and there were 4 replicate pens per treatment. Pullet placement was performed at 15 weeks of age and the trial lasted until 50 weeks of age. Equal dry matter of substrates was provided across treatments.

Recorded production parameters such as mortality, apparent feed intake, egg production, and feed conversion ratio, egg quality, and proportion of cracked egg were not affected by litter substrate. High mortality and culls were recorded across treatments due to a general incidence of injurious pecking. However, culls due to pecking injuries were lowest in the peatmix compared to the biochar treatment, suggesting a reduced level of injurious pecking among peatmix hens. Welfare indicators such as litter and perch usage, foraging, fearfulness, and integument conditions were not affected by litter substrate. However, lying in the litter was observed less in peatmix compared to wood shavings.

Furthermore, the proportion of cracked eggs increased with hen age (P<0.001) whereas the proportion of dirty eggs decreased with hen age (P<0.001). Egg albumen height and haugh unit decreased with increasing storage time and hen age (P<0.001). The results of this study did not show any major effect of litter substrate on production performance and welfare in laying hens. This may indicate that the hens adapted in a similar manner in terms of litter usage when litter substrates were provided as a single choice materials.

## Introduction

In poultry facilities, a good quality litter bedding is crucial for droppings dilution, moisture absorption, and thermal insulation to ensure comfortable conditions for birds (Shepherd et al. 2017). It also enriches the birds’ environment as a suitable medium to support the expression of natural behaviors such as scratching, dustbathing, and foraging, thereby improving their general welfare (Scholz et al. 2010; Shepherd et al. 2017). A lack of or insufficient access to litter can lead to severe feather pecking and consequently poor plumage conditions, mortality, increased energy demand, and reduced egg production (Rodenburg et al. 2013; Schreiter et al. 2019). The choice of litter substrate is predominantly influenced by availability and cost. However, substrate characteristics such as composition, particles size, and moisture content are also important factors that can impact litter quality and thus poultry welfare and performance (Scholz et al. 2010; Villagrá et al. 2011; Brink et al. 2022; Holt et al. 2023).

Wood shavings is one of the most frequently used litter substrates for poultry due to its availability (Sanotra et al. 1995; Villagrá et al. 2014; Brink et al. 2022). With the right structure, it can possess good water- holding and releasing capacity, and it also produces less dust compared to other substrates such as peat (Munir et al. 2019). Peat, on the other hand, is preferred more by birds in terms of fulfilling the need for dustbathing or foraging, likely because of its small particle sizes (De Jong et al. 2007; Villagrá et al. 2014; Holt et al. 2023). Moreover, it can rapidly absorb and release moisture, and its low pH may neutralize litter ammonia (Shepherd et al. 2017), resultingly affecting litter related behaviors (Kristensen et al. 2000). Using peat in animal stables also facilitates manure management for soil application (O Englund personal communication, June 13, 2024). Nevertheless, its continuous usage as litter substrate necessitates a strong justification since its extraction is associated with greenhouse gas emissions (Väisänen et al. 2013).

Biochar, a potential litter substrate, is a carbon-rich material produced from the pyrolysis of organic material. Due to its high porosity and sorption capacity, it is used as a bulking agent in manure and to reduce ammonia volatilization during composting (Janczak et al. 2017; Kalus et al. 2019). Like peat, using biochar as a litter substrate may pose a risk of increased dustiness which can negatively affect hen and egg cleanliness. However, this risk could possibly be mitigated if it is used as a litter amendment or a co-substrate. Litter amendment with biochar has been reported to reduce pen odor (Gerlach & Schmidt 2012). Another potential litter amendment strategy is the use of microbial additive. A selected blend of microbes may improve the quality of the litter, by different actions leading to reduced ammonia volatilization (Gutarowska et al. 2014; Borowski et al. 2017), and in turn improve the attractiveness for laying hens to use the litter.

In egg production, performance parameters such as feed intake, laying rate, feed conversion ratio (FCR), and egg quality are key parameters of interest but there is insufficient information on the influence of different litter substrates on them. Since hens’ litter related behavior varies between litter types (De Jong et al. 2007; Skånberg et al. 2021), it is possible that their reactions to different litter substrates may translate into differences in production performance. For instance, variations in the foraging behavior of birds may lead to differences in severe feather pecking behavior (Huber-Eicher & Wechsler 1998), and this can resultingly affect mortality and/or production performance. Furthermore, given that birds are prone to ingesting litter material (Hetland & Svihus 2007), the possible effect on hens’ productivity cannot be ruled out especially when litter is amended with novel materials. There could be a positive effect through improved gut functions resulting from eubiotic mechanisms in the gut, or a negative effect through dilution of nutrient intake leading to increased feed intake or reduced nutrient utilization.

While most litter studies with laying hens have primarily been preference tests of multiple litter substrates in relation to welfare, knowledge of the corresponding impact of litter substrate on performance and egg quality is limited. Furthermore, despite the application of biochar in composting poultry manure, there is limited knowledge on its direct usage as a litter substrate in poultry houses. The aim of this study was to investigate the impact of four main litter strategies on various welfare parameters and productivity in laying hens. The strategies were pure wood shavings, wood shavings mixed with peat, wood shavings amended with biochar, and wood shavings amended with microbial additive.

## Materials and methods

### Animals, housing, and diet

The experiment was approved by the Animal Ethics Committee of Uppsala region in Sweden (Decision number 5.8.18-20113/2022). The experiment was conducted between 24^th^ January and 25^th^ September 2023 in the laying hen facility of the Swedish Livestock Research Centre, Uppsala, Sweden.

**Fig. 1:**
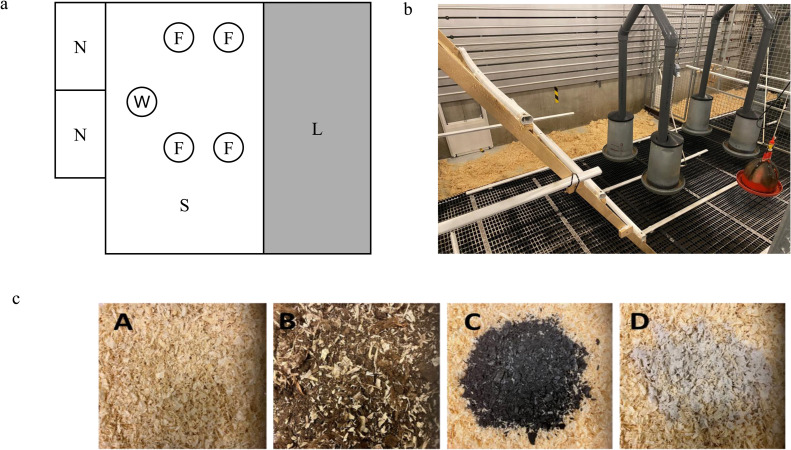
1a. Schematic of a pen. Total area of a pen was 12.9 m^2^ including a litter area (L) (1.32 × 3.56 m) and a slatted floor area (S) (2.30 × 3.56 m) with 2 group nests (N) (1.15 × 0.46 m each), a bell drinker (W), and 4 round feeders (F). 1b. Photo of a pen showing the slatted and litter areas. The walls of adjacent pens were covered with white polythene sheets (not shown in his photo). 1c. Litter treatments. Consist of four litter substrates; A is pure wood shavings; B is a mixture of peat and wood shavings (Peatmix); C is a combination of biochar and wood shavings; D is wood shavings with microbial additive.

A total of 1600 Bovan White pullets with intact beaks bought from a commercial farm were used in the experiment. Placement of pullets was carried out at 15 weeks of age and the trial lasted until 50 weeks of age, which included a 3-week adaptation period. Pullets were placed in groups of 100 in each of the 16 identical pens in the same building. There were 2 rows of 8 adjoining pens separated by an aisle. The wire meshes of adjoining pens were covered with white polythene sheets to prevent interactions between adjacent groups. Each pen had a traditional floor housing system with a total floor area of 12.9 m^2^ of which 4.7 m^2^ (1.32 × 3.56 m) was a litter area and the remaining 8.2 m^2^ (2.30 × 3.56 m) was a raised slatted floor which had 1 bell drinker, 4 feeders, and access to 2 group nests, each with a dimension of 1.15 × 0.46 m (Fig. 1a and 1b). All pens were equipped with a similar number of perches (> 15 cm/ hen) in the slatted space. Automated floor scrappers below the slatted floor were used to remove manure once a day. Eggs were manually collected each day from the egg belts located outside, behind the nests. The hens were initially given 9 h of light per day at placement which gradually increased to 15 h (06:00- 21:00) at 22 weeks of age until the end of the experiment. The stable was equipped with an automated ventilation and heating system to maintain similar internal climatic conditions in pens with the aim of keeping the temperature at around 20°C. Humidity ranged between 18% and 55%.

Feed and water were provided ad libitum. All hens received the same standard commercial layer diet, fed as crumbles in three phases with ME contents of approximately 11.3 MJ/kg. Phase 1 was 16 to 29 weeks of age with content calculated of 17.2% CP and 3.7% Ca. Phase 2 was 30 to 39 weeks of age with a content of 16.8% CP and 3.8% Ca. Phase 3 was 40 to 50 weeks of age with a content of 16.5% CP and 3.8% Ca (Lantmännen, Falkenberg). The hens received extra coarsely ground oyster shells daily (130 g/pen/day) from 24 weeks of age and a bale of alfalfa from 32 weeks of age until the end of the experiment. These were to serve as additional environmental enrichments to reduce pecking incidence among the flocks.

### Experimental design

Treatments were randomly assigned to each of the 16 pens containing 100 pullets. There were 4 replicates per treatment. The treatments were 4 different litter substrates, namely: wood shavings, peatmix, biochar, and microbial additive (Fig. 1c). The wood shavings treatment consisted of 100% wood shavings. The peatmix treatment was a pre-mixed material of 20% wood shavings and 80% peat. The biochar treatment was a combination of wood shavings and biochar (Supplementary Table 1). The microbial additive treatment contained wood shavings and a compost additive, Bactériolit® (SOBAC, Lioujas, France), comprised of a complex of microorganisms, natural minerals, and selected composted plants. The wood shavings and peatmix were supplied by YesBox (YesBox, Skutskär Sweden). The biochar was European Biochar Certificate (EBC) feed grade supplied by Oplandske Bioenergi (Oplandske Bioenergi AS, Biri, Norway).

All pens initially received the same amount of wood shavings (1285 g DM/ pen) during the 3-week adaptation period until treatments were assigned at 18 weeks of age. Thereafter, weekly provision was adapted after the different litter beddings were assessed. Replenishment was based on a minimum litter depth of < 0.5 cm covering > 50% of the litter area. Equal DM of substrates were provided across treatments. However, weekly provision per pen varied from 700 g to 2800 g DM. The DM contents of wood shavings and peatmix were 90% and 50% respectively. Initial DM content of biochar was about 86% and this was moistened to 70% to reduce dustiness. Weekly biochar quantity was maintained at 700 g DM. Supplementary Table 1 shows the four main options for the weekly litter replenishment and the explanations for our choice of inclusion levels of peatmix, biochar and microbial additive. Each pen received a total substrate DM of about 70 kg over the 35-week experimental period. Bactériolit® was spread on top of the wood shavings at a dosage of 1 g/hen/week, correcting for mortality.

To obtain an indication of the substrates’ initial characteristics, samples of biochar, peatmix, and wood shavings kept at room temperature were evaluated for their water holding capacity (WHC) and particle sizes. WHC was assessed using a method adapted from Shepherd et al. (2017). Particle size distribution was assessed with a sieve analysis machine (JEL, Engelsmann). At the end of the trial, the total litter in each pen was weighed and samples were analyzed for DM. Examples of the appearance of the litter beddings at age 49 weeks are provided in supplementary figure 1.

### Data collection

***Production Performance.*** Mortalities and culls were recorded daily. Mortalities refer to hens that were found dead and culls refer to hens that were euthanized or excluded from the experiment mainly due to severe pecking injuries. Visual assessment of injurious pecking was done by trained farm technicians according to the ethical application protocol. Hens that sustained very severe injuries and were unlikely to survive if they remained in the experiment were either euthanized or moved permanently to a recovery pen. In case of slight skin damage, the hen was kept in the experiment and treated using Anti-cannibalism Spray No Bite (Albert Kerbl GmbH, Buchbach, Germany). The total number of eggs laid were recorded daily. Floor eggs, which are eggs laid on the slatted floor plus eggs laid in the litter, were also recorded. Egg weight was recorded weekly. Residual feed per pen was recorded on a 28-day basis and from this, the average daily feed intake per hen was calculated correcting for mortality and culls. In this study feed intake was referred to as apparent feed intake as we could not correct for feed losses. Laying rate was calculated by dividing the number of eggs laid by the number of hens in the pen corrected for mortality and culls. Egg mass (g/hen) was calculated by multiplying percentage of egg production by average egg weight. Feed conversion ratio (FCR) was calculated as the ratio of average daily apparent feed intake and egg mass. Production performance data from 20 to 50 weeks of age were expressed as mean values per pen and period for statistical analysis. A period was based on a 28-day interval and there were 8 periods from 20 to 50 weeks of age.

***Cracked and Dirty Eggs.*** At 32, 38, and 49 weeks of age, eggs collected on 3 consecutive days of each week were examined to determine if they were cracked or dirty using a candling machine. Proportions of cracked and dirty eggs were calculated for statistical analysis.

***Egg Quality*.** To assess egg quality, 10 clean eggs laid in the nest without any visible cracks were collected from each pen at 39, 43, and 50 weeks of age, and egg weight, shell breaking strength, shell thickness, albumen height, and albumen DM were measured. Due to the large number of collected eggs, measurements were taken over a 3-day period in each week, and the eggs were stored at a temperature of 4°C until the final days of analysis. Eggshell breaking strength was measured with an Egg Force Reader (EF 0423 Orka Food Tech. Israel). The content of the broken egg was poured on a flat surface (glass), and the albumen height was measured about 0.5 cm from the edge of the yolk with a tripod micrometer (S-6428 Ames Co. Waltham, Mass). The albumen was separated from the yolk, and the weight was recorded. The albumen was placed in the oven at 60°C overnight and subsequently dried again at 103°C for 24 hours. The weight of the dried albumen was recorded, and the DM was calculated. Three pieces of the eggs’ shell without the shell membrane taken from the equatorial area were measured for egg thickness with a digital micrometer (ID-C112B, Mitutoyo Corp. Japan). Haugh Units (HU) was calculated as 100 x log (H – 1.7W^0.37^ + 7.57), where H is albumen height (mm) and W is egg weight (g) (Silversides & Villeneuve 1994).

***Integument and Keel Bone Scoring.*** At 36 and 50 weeks of age, 20 hens per pen were randomly selected for integument and keel bone scoring based on the protocol described by Tauson et al. (2005). The hens were weighed and scored on a scale of 1 (worst) to 4 (best) for feather covers on 6 body parts: the neck, breast, cloaca, back, wings, and tail. Similarly, they were scored on feather cleanliness, peck injury on comb, peck injury on the rear, bumblefoot, and keel bone deviation. Scoring of keel bone deviation was performed using the palpation technique on a 4-point scale (1: severe, 2: moderate, 3: slight, 4: no deviation). All scoring was carried out by the same trained individual. The feather cover scores of the 6 body parts were added together, generating a general plumage condition score ranging from 6 (worst) to 24 (best).

***Birds’ Activity*.** To evaluate the rate of use of litter area and performance of litter related activities, direct behavioral observations were performed once per week by two trained observers on different occasions due to personnel availability: the first one from 33-46 weeks of age and the second one from 47-50 weeks of age. The observations occurred one day after the weekly litter replenishments. Before replacing the first observer, both observers performed the same observation concurrently and their results were similar. An observation involved a scan of each pen to count and note perching and litter related behaviors specified in the ethogram (Table 1). There were two observation periods during the daytime: morning (10:30 and 11:30) and afternoon (13:30 and 14:30). Before each observation commenced, the observer walked along the aisle outside the pens for 5 min to accustom the hens to their human presence. During the observations, the observer stood by the entrance of each pen. For practical purposes, hens that were simply standing and/or moving in the litter area were observed as one behavior and accordingly described as standing/moving. Likewise, dustbathing and/or resting were described as lying due to the challenge of differentiating between the two activities from a distance within a short period. The proportion of hens on the perches and the proportion of hens in the litter were calculated based on total number of hens in the pen correcting for mortalities and culls. The remaining hens, mostly on the slatted floor were not counted. The proportions of hens that were standing, preening, foraging, and lying in the litter were calculated based on the total number in the litter area.

**Table 1:** Definitions of observed behaviours of hens in the pens.

Behavior	Definition
Preening	The hen's beak moves and touches its own body and carries out pecking or nibbling.
Foraging	The hen is pecking the litter while standing, walking, or scratching the ground.
Lying	The hen performs repeated behavioral moves including scratching, pecking the floor, shaking its wings, and feather ruffling while lying down (sometimes lying on the side with one leg up) or just squatting /lying down.
Standing/moving	The hen is standing without performing any of the other defined behaviors or it is moving from one location to another driven by wings and/or leg movements.

***Novel Object Test*.** At 41, 45, and 49 weeks of age, fear level of hens was assessed using the novel object (NO) (Welfare Quality, 2009) with small adjustments. This was performed by the same individual each week between 14:00 to 17:00. Before the start of each test, the individual walked along the aisle outside the pens for 10 min to accustom the hens to their human presence. The individual then slowly entered each pen, sat down on the edge of the slatted area, and waited for 3 min to again accustom the hens to the human presence. A multi-colored banded stick, 30 cm long with a diameter of 2 cm, was placed in the middle of the litter area. The individual then took a few steps back, about 1.5 m away from the NO, and immediately counted the number of hens within 1 hen length (ca. 35 cm) of the NO every 10 s for a total of 2 min. The mean number of hens per recording was used to calculate the proportion of the flock that came within 1 hen length of the NO. Low values indicated a high avoidance or fearfulness level.

### Statistical analysis

General linear models (GLM) were fitted for overall mortality and cull data. A linear mixed model (LME) with restricted maximum likelihood (REML) estimation was fitted using the nlme-package (Pinheiro et al., 2023) for production performance, egg quality, hen weight, feather cover, and NO test data. Model assumptions were checked visually using residual plots (q-q plots and histograms)

Generalized linear mixed models (GLMM) with a negative binomial distribution were used to analyze the rate of activities for the litter behavior data using the package glmmTMB (Brooks et al., 2017). Feather cleanliness, feet cleanliness, peck injury, and bumble foot data were transformed into binary outcomes (0 for scores ≤ 2 and 1 for scores ≥ 4) and were analyzed with a GLMM assuming a binomial distribution (package glmmTMB; Brooks et al., 2017). Model assumptions were checked using the package DHARMa (Hartig, 2022). Data on keel bone deviation and preening did not have enough variability for statistical analysis.

Measurements were repeated over time in all mixed models. All repeated measurements were analyzed using a mixed model of treatment (4 litter substrates), hen age/period (2 to 18 time points depending on the measurement), and their interactions as fixed effects, and pen as a random effect. For egg quality data, day of analysis (3 storage times) was included in the model to account for the influence of storage time. For behavior data, observation period and treatment x observation period interaction was included. Treatment x age interaction was excluded. For the repeated measures analysis an error term using a continuous autoregressive correlation structure was used for all production performance variables. A general correlation structure was used in the analysis NO test, cracked eggs, and dirty eggs data.

Pairwise comparison of least square means was adjusted for using Tukey–Kramer adjustment for multiple comparisons (package emmeans; Lenth 2024). A statistically significant difference was considered at P < 0.05 and a tendency at 0.05≤ P ≤ 0.1. Data analysis was performed in R software (version 4.3.1; R Core Team, 2023).

## Results

### Mortality and production performance

**Fig. 2:**
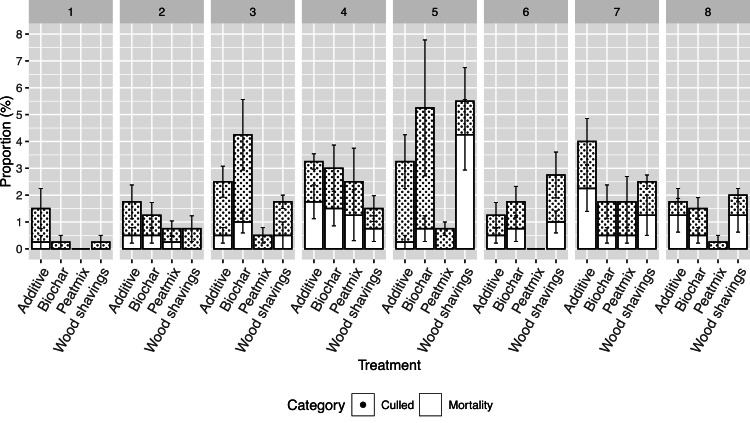
Effect of litter strategies on mortality and proportion of culled hens from period 1 to 8. A period was based on a 28-day (4 week) interval. There were 8 periods from 20 to 50 weeks of age.

At the end of the experimental period, 95 hens had died, and 152 hens had been culled. Of the culls, 45 hens were euthanized, and 107 hens were excluded from the experiment and transferred to a recovery pen. About 91% of the deaths and culls were a result of injurious pecking of the feet, rear, head, and neck. Treatment did not affect mortality (Table 2). However, there was considerable within-treatment variability. There was a treatment effect on the proportion of culled hens with a significant difference between peatmix (3.5%) and biochar (13.5%). Numerically, mortality and cull rates were the lowest in period 1 and the highest in period 5 (Fig. 2). Peatmix had numerically lower mortality and cull rates in most of the production periods (Fig. 2).

**Table 2:** Effect of litter strategies on mortality and proportion of culled hens from 20 to 50 weeks of age. Values presented are least square means.

Treatment^1^	Mortality^2^ (%)	Culls^3^ (%)
Wood Shavings	9.0	8.0^ab^
Peatmix	2.0	4.5^b^
Biochar	5.5	13.5^a^
Additive	7.3	12.0^ab^
SE	1.9	2.1
P value	0.107	0.046

1 Wood shavings- treatment was pure wood shavings; Peatmix- treatment was a mixture of 80% peat and 20% wood shavings; Biochar- treatment was a combination of biochar and wood shavings; Additive- treatment was wood shavings with microbial additive.

2 Proportion of hens that were found dead in the pen.

3 Proportion of hens that were euthanized or permanently removed from the experiment due to severe pecking injuries and health issues. SE is Standard error of least square means.

a,b,c Means a lacking a common superscript are significantly different (P<0.05).

As shown in Table 3, apart from average egg weight which was affected by treatment, there was no effect of treatment on apparent feed intake, egg mass, and FCR, proportion of litter eggs and proportion of floor eggs. However, the difference in egg weight was not evident after the Tukey-Kramer adjustment for multiple comparison. All production performance results including apparent feed intake, egg weight, egg mass, laying percentage, FCR, and floor (slat + litter) eggs were affected by period apart from the proportion of litter eggs which showed a tendency to differ. There was treatment x period interaction effect on apparent feed intake. Microbial additive hens had a higher apparent feed intake compared to biochar hens in period 7 (133 g > 125 g, P = 0.046) and period 8 (128 g > 118 g, P = 0.025), and a higher apparent feed intake than wood shavings hens in period 8 (128 g > 118 g, P = 0.025).

**Table 3:** Effect of litter strategies on production performance and proportion of floor eggs among laying hens from 20 to 50 weeks of age. Values presented are least square means.

Item	ADFI^2^	Egg weight	Egg mass	Laying^3^	FCR^4^	Litter^5^ egg	Floor^6^ egg
	(g/hen/d)	(g)	(g)	rate (%)	(g/g)	(%)	(%)
Treatment^1^							
Wood Shavings	121	60.9	56.9	92.6	2.25	0.15	0.38
Peatmix	122	61.3	57.0	92.2	2.29	0.20	0.51
Biochar	120	60.5	56.7	92.8	2.26	0.20	0.43
Additive	124	60.5	56.8	93.2	2.31	0.09	0.42
SE^7^	1.4	0.2	0.2	0.3	0.04	0.08	0.10
Period^8^							
1	126±0.9^b^	51.5± 0.2^e^	30.1±0.3^e^	58.4± 0.6^b^	4.20± 0.06^a^	0.32± 0.07	1.24± 0.10^a^
2	133±0.9^a^	57.6± 0.2^d^	56.0± 0.3^d^	97.2± 0.4^a^	2.38± 0.02^b^	0.16± 0.05	0.43± 0.06^b^
3	115±0.6^d^	61.0± 0.2^c^	59.7± 0.2 ^c^	97.8± 0.2^a^	1.93± 0.02^d^	0.16± 0.04	0.36± 0.06^bc^
4	116±0.7^d^	60.8± 0.2^c^	59.4± 0.2^c^	97.9± 0.2^a^	1.95± 0.02^d^	0.15± 0.05	0.31± 0.06^bc^
5	115±0.8^d^	63.7± 0.1^b^	62.4± 0.1^ab^	97.9± 0.1^a^	1.85± 0.02^e^	0.15± 0.04	0.32± 0.05^bc^
6	120±1.5^c^	63.8± 0.2^ab^	62.3± 0.2 ^ab^	97.7± 0.2^a^	1.92± 0.02^d^	0.13± 0.06	0.31± 0.07^bc^
7	128±1.0^b^	64.2± 0.1^a^	62.6± 0.1 ^a^	97.6± 0.1^a^	2.05± 0.02^c^	0.11± 0.04	0.27± 0.05^c^
8	121±1.1^c^	63.8± 0.1^b^	62.1± 0.1^b^	97.3± 0.2^a^	1.95± 0.02^d^	0.10± 0.06	0.26± 0.07^bc^
P-value							
Treatment (T)	0.561	0.031*	0.139	0.054	0.432	0.577	0.353
Period (P)	<0.001	<0.001	<0.001	<0.001	<0.001	0.051	<0.001
T x P	0.020	0.110	0.103	0.055	0.068	0.174	0.154

1 Wood shavings- treatment was pure wood shavings; Peatmix- treatment was a mixture of 80% peat and 20% wood shavings; Biochar- treatment was a combination of biochar and wood shavings; Additive- treatment was wood shavings with microbial additive. n = 32 per treatment.

2 Average daily apparent feed intake.

3 Based on the number of hens in the pen corrected for mortality and culls.

4 FCR- feed conversion ratio.

5 Proportion of eggs laid in only the litter area.

6 Proportion of eggs laid in both slatted and litter areas.

7 A period was based on a 28-day interval. There were 8 periods from 20 to 50 weeks of age.

⁎ Difference in egg weights was not evident after the Tukey-Kramer adjustment for multiple comparisons. ^a,b,c^ Means within a column of a section lacking a common superscript are significantly different (P<0.05).

### Cracked and dirty eggs

Supplementary table 2 shows the results of the proportions of cracked or dirty eggs. Treatment had no effect on the proportion of cracked eggs. There was a treatment effect on the proportion of dirty eggs, with peatmix and biochar having higher values than wood shavings and microbial additive, however, this was not evident in the Tukey-Kramer adjustment for multiple comparisons. The proportion of cracked eggs increased with age whereas proportion of dirty eggs decreased with age. There was no treatment x age interaction effect on the proportion of cracked or dirty eggs.

### Egg quality

Treatment had no effect on egg weight, egg breaking strength, shell thickness, albumen height, albumen DM, and HU (Supplementary table 3). However, there was an effect of hen age on all the egg qualities’ parameters except for egg breaking strength. Albumen height, HU, and albumen DM decreased with increasing storage time (Day) and age. However, storage time effect on albumen DM was not evident in the Tukey-Kramer adjustment for multiple comparisons. There was no treatment x age effect on any of the parameters.

### Hen weight and integument score

As shown in supplementary table 4, treatment had no effect on hen weight and integument scores. Hen weight, feet cleanliness, and peck injury on the comb improved with hen age whereas feather cover and peck injury at the rear worsened with hen age. However, hen age did not affect feather cleanliness and bumblefoot. There was a treatment x age interaction effect on feather cover and peck injury at the rear, but the Tukey-Kramer post hoc test did not reveal difference between any interaction comparisons of relevance. Keel bone deviation in the hens was similar across treatments with mean scores of approximately 3.9 (results not shown).

### Perching and litter related activities

There was no effect of treatment on both the proportions of hens on perches and in the litter area (Table 4). Similarly, the proportions of hens in the litter area that were standing/moving or foraging were not affected by treatment. Lying was similar in peatmix and biochar but observed less in peatmix than in wood shavings and microbial additive. Hen age affected all parameters in an unpredictable manner. A greater number of hens were observed in the litter area in the afternoon than in the morning, and more foraging or lying was performed in the afternoon than in the morning. Conversely, more standing/moving in the litter was observed in the morning than in the afternoon. There was a treatment x observation period interaction effect on the proportion of hens that used the litter, but the Tukey-Kramer post hoc test did not reveal difference between any interaction comparisons of relevance. Preening was noted less, with mean proportion ranging between 0.6% and 0.7% across treatments (results not shown). Supplementary table 5 shows space occupancy by the hens. Average available space per hen ranged from 1627-1864 cm^2^/ hen on the slatted floor and 2562 - 3135 cm^2^/ hen in the litter area.

**Table 4:** Effect of litter strategies on the percentage of hens using the perches and the litter area. Values presented are least square means with their standard errors.

		On perch^4^	In litter^4^	Standing/	Foraging^5^	Lying
Item		(%)	(%)	Moving^5^ (%)	(%)	(%)
Treatment^1^						
Wood Shavings		22.5 ± 1.4	19.6 ± 0.6	25.7 ± 1.1	40.2 ± 1.4	18.3 ± 1.0^a^
Peatmix		23.5 ± 1.4	20.4 ± 0.6	27.0 ± 1.1	43.6 ± 1.4	13.2 ± 0.8^b^
Biochar		24.8 ± 1.5	19.4 ± 0.6	25.8 ± 1.1	42.6 ± 1.5	16.0 ± 1.0^ab^
Additive		25.7 ± 1.6	19.9 ± 0.7	25.5 ± 1.1	42.2 ± 1.4	17.6 ± 1.0^a^
Morning^2^						
Wood Shavings		22.9 ± 1.5	18.8 ± 0.7	28.3 ± 1.7	39.2 ± 2.0	15.6 ± 1.3
Peatmix		23.3 ± 1.5	20.1 ± 0.8	28.0 ± 1.5	42.5 ± 1.9	11.5 ± 1.0
Biochar		25.3 ± 1.6	17.7 ± 0.7	27.1 ± 1.7	40.4 ± 2.1	14.4 ± 1.3
Additive		25.3 ± 1.6	18.2 ± 0.7	28.1 ± 1.7	40.8 ± 2.1	14.9 ± 1.3
Afternoon^3^						
Wood Shavings		22.0 ± 1.4	20.4 ± 0.8	23.3 ± 1.4	41.3 ± 1.9	21.5 ± 1.5
Peatmix		23.8 ± 1.5	20.7 ± 0.8	26.1 ± 1.5	44.7 ± 1.9	15.1 ± 1.2
Biochar		24.4 ± 1.6	21.2 ± 0.8	24.5 ± 1.5	44.8 ± 2.0	17.8 ± 1.3
Additive		26.0 ± 1.7	21.8 ± 0.8	23.2 ± 1.4	43.6 ± 2.0	20.9 ± 1.4
Observation period						
Morning		24.2 ± 0.8	18.7 ± 0.4^b^	27.9 ± 0.9^a^	40.7 ± 1.0^b^	14.0 ± 0.6^b^
Afternoon		24.0 ± 0.8	21.0 ± 0.4^a^	24.2 ± 0.7^b^	43.6 ± 1.0^a^	18.6 ± 0.7^a^
P-value						
Treatment (T)		0.422	0.724	0.730	0.356	<0.001
Age^6^		<0.001	<0.001	<0.001	<0.001	<0.001
Observation period(P)	0.794		<0.001	0.001	0.042	<0.001
T x P		0.480	0.025	0.624	0.944	0.835

1 Overall treatment means for the entire observation period. ^1^Wood shavings- treatment was pure wood shavings; Peatmix- treatment was a mixture of 80% peat and 20% wood shavings; Biochar- treatment was a combination of biochar and wood shavings (Table 1); Additive- treatment was wood shavings with microbial additive.

2 Treatment means of morning observations.

3 Treatment means of afternoon observations.

4 Proportions are based on the total number of hens in the pen during behavior observations.

5 Proportions are based on the total number of hens in the litter during behavior observations.

6 Comprised of 18 time points from 33 to 50 weeks of age. ^abc^ Means within a column of a section lacking a common superscript are significantly different (P<0.05).

### Novel object (NO) test

The NO test did not show that substrate type affected the fear level of hens (SEM= 0.65, P = 0.242). The average proportion of hens within one hen length of the NO was 10.5% for wood shavings, 10.4% for peatmix, 10.9% for biochar, and 11.0% for microbial additive. The number of hens within one hen length decreased with age (SEM= 0.35, P < 0.001). The average proportion of hens within one hen length of the NO were 11.9, 10.4, and 9.6 % at 41, 45, and 49 weeks of age, respectively. There was no treatment x age interaction effect.

### Substrate and litter properties

Peatmix had the highest WHC (418.8%) and biochar the lowest (234.9%) at room temperature. Supplementary table 6 shows the WHC of the three litter materials and the corresponding DM contents and particle size distribution at room temperature. The proportion of fine particles (< 0.4 mm) was the highest in peatmix while the proportion of coarse particles (> 4 mm) was the highest in wood shavings. Biochar had the greatest proportion of intermediate particles sizes (0.4- 4 mm). The average weights of the remaining litter in the pens at the end of the experiment were similar across all treatments, but there was large variability within treatment replicates. Peatmix had the lowest DM content across all treatments. The litter weights were 11.8 kg (69.5% DM) for wood shavings treatment, 13.2 kg (59.8% DM) for peatmix treatment, 11.9 kg (72.9% DM) for biochar treatment, and 13.1 kg (74.4% DM) for microbial additive treatment. Average litter depths ranged from 0.5 to 5 cm in the pens during the experiment period.

## Discussion

Mortalities and culls in this study were predominantly due to injurious pecking on the feet, rear, neck, or head areas. The provision of environmental enrichments such as alfalfa bale and oyster shells does not seem to have significantly mitigated the general incidence of injurious pecking. It may appear that there was an added benefit of peatmix in reducing the injurious pecking incidence given the generally lower mortality and culls among peatmix hens compared to the other treatments. This presumption, however, is not supported by our results on foraging behavior as well as the integument assessment considering their likely relationships with injurious or feather pecking (Huber-Eicher & Wechsler 1998; Tauson et al. 2005). The cause of injurious pecking can be multifaceted, and the high levels observed in this study could be due to stress associated with the transition of the pullets from a relatively complex rearing environment (aviary) to a traditional floor housing for our experiment. Indeed, studies show that deaths caused by injurious pecking may occur due to stress arising from transporting, relocation, and mixing of birds (Van De Weerd & Elson 2006; Colson et al. 2008; Cronin et al. 2018). Furthermore, the possibility that the group size of 100 hens influenced the general pecking incidence in this study cannot be excluded. Flock size is an important factor that can influence the occurrence of feather pecking or aggression. Aggressive pecking increased with increased flock size in a study where hens were kept in groups of 15, 30, 60, and 120 with the same stocking density in deep litter (Bilčík & Keeling 2000).

Hens’ integument conditions were assessed as welfare indicators in relation to substrate type. The comparable integument scores suggest similar hygiene and feather pecking behavior across treatments. Hens in biochar were expected to have dirtier plumage than those in the other substrates, but feather and feet cleanliness scoring did not reveal any differences despite biochar hens appearing somewhat greyish, which was not considered a hygiene issue. The biochar dosage used in this study (1g DM/hen/d plus wood shavings) and the initial moistening might have helped reduce the amount of biochar dust in the air. Previous studies with broilers did not detect integument discolorations when biochar was used as litter amendment (Linhoss et al. 2019; Ritz et al. 2011) likely because, compared to layers, broilers dustbathe less due to their body weight and conformation (Dawson et al. 2021; Nicol et al. 2024). Regmi et al. (2018) and De Jong et al. (2013) reported that the type of litter substrate did not influence feather damage in laying hens. However, other studies had suggested that the form of substrate as a foraging material, rather than a dustbathing material, can influence feather pecking behavior (Huber-Eicher & Wechsler 1997, 1998).

At the end of our trial, less than 14% of the total substrate added (ca.70 kg DM) to the pens remained in the litter area. This was mainly because of litter material falling through the slatted area when the birds returned from using the litter area, indicating a high level of litter activity by the birds in this study. Even though litter replenishment was carried out frequently, this was not initially intended as it was expected that after the initial litter provisions, the substrates and manure would build up over time like in commercial settings where little or no litter replenishment is done during a production cycle.

The birds may have adapted their litter behavior to the different substrates in a similar manner, resulting in the seemingly comparable levels of standing/moving and foraging behavior in the litter. De Jong et al. (2007) suggested that hens may not have a strong substrate preference for foraging. Thus, a hen’s preference for one substrate over another regarding performance of litter related activities is perhaps only evident when they are given the opportunity to choose (Monckton et al. 2020; Skånberg et al. 2021). This study, using a single litter approach, sought to explore hens’ acceptance of a litter substrate in terms of litter usage, and the effects on other welfare and production parameters. Therefore, it is not directly comparable with previous studies as these have been largely conducted using multi-choice approaches (resource choice and consumer demand) to assess hens’ substrate preference.

In the present study, both dustbathing and resting behavior in the litter were described as lying due to the difficulty in differentiating between the two activities with our observation method. Lying behavior was lowest in peatmix contrary to previous findings that peat is the most preferred material for dustbathing (De Jong et al. 2007; Monckton et al. 2020; Skånberg et al. 2021). However, if hens with free access to litter, dustbathed every second day (Vestergaard 1982) then it is possible that hens in peatmix satisfied their need for dustbathing on the first day of litter replenishment and therefore dustbathed less the next day during the behavior observations. It is also worth noting that data on hens’ litter behavior was collected a bit later in the present study (age 33 weeks) due to technical challenges. Therefore, the possibility of missing out on insight into earlier litter usage cannot be overlooked. Perhaps data on litter behaviors earlier in the experiment would have provided a broader scope for the evaluation of treatment -age interrelationship on substrates acceptance and utilization from the onset. The hens in this study exhibited litter behavior more in the afternoon than in the morning, similar to the findings of Mishra et al. (2005) and Campbell et al. (2016). This is likely because they are more involved in other activities such as egg laying and feeding in the morning.

Although ammonia measurement was not a focus of the present study, we presumed that litter substrate might influence perch usage if the hens tried to avoid the litter area by using elevated areas due to their aversion to substances such as ammonia or dust (Kristensen et al. 2000). However, substrate did not influence perching behavior, as the perches were often occupied regardless of the substrate type. The results show that the hens used the perches in line with their natural behavior, probably because they were accustomed to using elevated platforms from their rearing facility (aviary setting). Litter substrate did not affect hens’ fearfulness as assessed by the NO test. However, given the relationship between fear and severe feather pecking (Vestergaard et al. 1993; Uitdehaag et al. 2008), it is possible that the high pecking incidence in our study might have masked any effect of litter substrate on fear. The hens in this study appeared to become more fearful with age, similar to the findings of Alm et al. (2015). In contrast, Hocking et al. (2001) and Albentosa et al. (2003) reported a decrease in fearfulness with age likely because of the method of fear assessment used in their studies. Unlike their studies, we used the same NO in the three assessment occasions, and it is possible that more hens in our study became familiar with the so-called NO, lost interest in it, and avoided it.

Results of litter effect on poultry performance have been inconsistent, with most showing no effects in broilers (Villagrá et al. 2011; Shepherd et al. 2017; Linhoss et al. 2019; Pereira et al. 2024). For broilers, the constant contact with litter may directly influence contact dermatitis and consequently feed utilization or health (De Jong et al. 2014:20). Layers, on the other hand, are not necessarily in continuous contact with the litter, so its negative effects as a result of contact dermatitis may not be as pronounced. Nevertheless, litter effects on other factors such as pecking behavior or nutrient intake and utilization which could possibly affect hens’ production performance were not evident in the present study.

For domestic hens, litter substrate may enhance both behavioral and physiological welfare through the mitigation of ‘negative’ welfare. However, enhanced behavioral welfare does not necessarily translate into increased production performance among high producing hens, especially when it does not lead to improvements in physical health. The egg production results in this study agree with the findings of Regmi et al. (2018) who reported that litter substrate did not influence egg production and the amount of floor eggs. Similarly, Hetland & Svihus (2007) reported no difference in egg production and egg weight between wood shavings and paper as litter substrates in enriched cages. However, feed intake was higher among the paper substrate hens probably due to inefficiencies associated with nutrient dilution resulting from paper consumption. The high apparent feed intake among microbial additive hens during the later periods of our experiment could be due to microbial influence in the gut through putative mechanisms such as energy harvesting or the production of metabolite which can influence the host’s appetite (Turnbaugh et al. 2006; Breton et al. 2016). As expected, period (age) affected all production parameters in this study. The unusually high apparent feed intake in periods 1 and 2 were due to feed spillages during feeding, as the automatic feeders were overfilled in the early periods. Over time, the hens became more accustomed to their new environment including the nest which resulted in less floor eggs.

The increase in cracked eggs as the hens aged was expected since age has a negative impact on eggshell qualities (Roberts et al. 2013). Moreover, shell thickness and egg breaking strength generally decreased with hen age, which could be attributed to changes in shell structural properties such as decreased mammillary density and increased size of crystal units in older hens (Benavides-Reyes et al. 2021). Our results show that egg albumen height, HU, and albumen DM are not affected by litter substrate but, rather, factors such as hen age and storage time, which is consistent with the findings of Hill & Hall (1980) and Silversides & Scott (2001). Albumen height and HU are indicators of egg freshness and over time, egg freshness decreases because material such as water and CO_2_ are lost through the shell (Heath 1977).

During egg candling, our visual assessment was quite stringent to include dust particles on eggs, therefore, high percentages of dirty eggs were recorded in this study. The high proportions of dirty eggs in peatmix and biochar treatments is probably due to their dustiness which was much more visible on the eggs from those treatments compared to wood shavings and microbial additive. The particle size analysis showed finer particles in peatmix and biochar than in wood shavings which could have posed a risk of dustiness particularly in peatmix (Shepherd et al, 2017). However, it is possible that their initially relatively low DM coupled with their combinations with wood shavings in this study helped to reduce the anticipated high levels of dust. Pure peat moss can absorb eight times its weight in water (Shepherd et al. 2017). Thus, the considerably high WHC of peatmix, influenced by its finer fractions, could have caused the relatively high litter moisture observed at the end of the trial.

Aside from providing comfortable conditions for hens, good quality litter must improve hens’ welfare by supporting the expression of litter related behaviors such as scratching, dustbathing, and foraging. The lack of substrate effect on litter utilization in the present study indicates the importance of free choice in the expression of litter related behaviors as demonstrated by several preference tests (De Jong et al. 2007; Skånberg, et al.2 021; Holt et al. 2023). Perhaps, without the freedom to choose, the hens in the present study utilized the litters provided to them in a very restricted way. Thus, comparable litter usages among treatments were observed in the present study. Further efforts to enhance hens’ welfare can consider the provision of more than one litter substrate to support the expression of their different natural behaviors.

## Conclusion

Both the type and quality of litter substrate have been reported as key factors in evaluating behavior, welfare, and performance of birds. However, in this study it could not be determined if litter substrate provided as a single choice can influence production performance and egg quality parameters among laying hens.

A combination of wood shavings and other substrates such as peat and biochar may help improve overall substrate properties and reduce the dustiness of biochar and peat. Thus, further studies are needed to investigate how peat or biochar may influence ammonia volatilization in hen houses. Such in-depth knowledge of their potentials as litter substrates will aid in their adoptability. Additionally, other factors such as the strain of hen and the flock cycle which may affect hens’ behavior and productivity could be considered in similar future studies.

## Disclosures

The authors confirm that there are no conflicts of interest.

## CRediT authorship contribution statement

**Nathaniel Nutsugah:** Writing – review & editing, Writing – original draft, Visualization, Methodology, Investigation, Formal analysis, Data curation. **Emma Ivarsson:** Writing – review & editing, Supervision, Investigation. **Anette Wichman:** Writing – review & editing, Methodology. **Helena Wall:** Writing – review & editing, Supervision, Project administration, Methodology, Investigation, Funding acquisition, Formal analysis, Data curation, Conceptualization.

## Disclosures

The authors declare the following financial interests/personal relationships which may be considered as potential competing interests:

Nathaniel Nutsugah reports financial support was provided by Swedish Research Council Formas. If there are other authors, they declare that they have no known competing financial interests or personal relationships that could have appeared to influence the work reported in this paper.

## Appendix Supplementary materials

Image, application 1

Image, application 2
