# Supplementary material for: Influence of litter substrates on production, egg quality, and welfare indicators in laying hens
Source: Poult Sci. 2025 Dec 5;105(1):106201. doi: 10.1016/j.psj.2025.106201 (PMC12771291; doi:10.1016/j.psj.2025.106201)
Supplement: Supplementary file 1 [file mmc1.pdf]

Supplementary figure 1

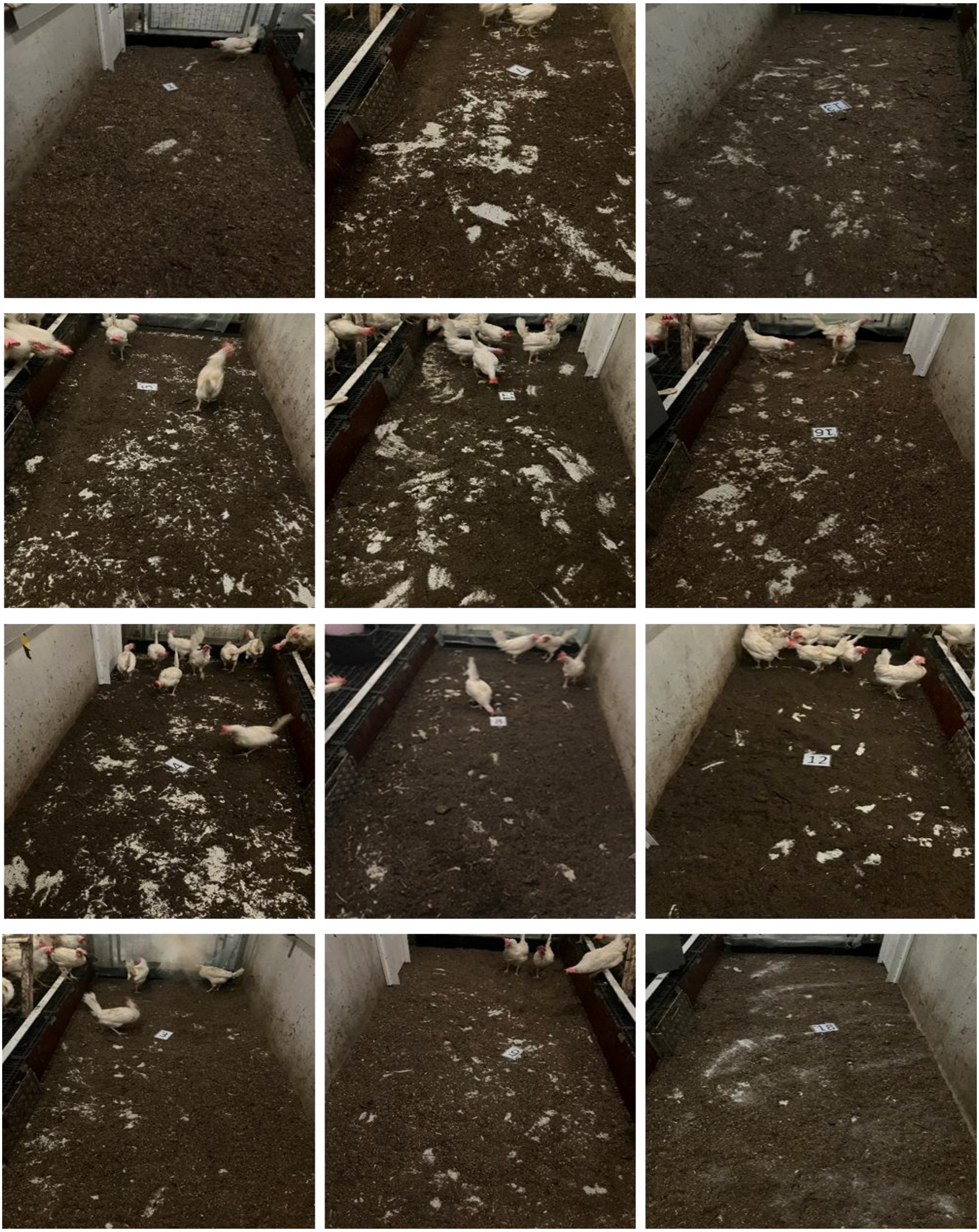

Some photos of the litters at age 49 weeks. Top row (pens 1, 7, 13) are wood shavings treatment. Second row (pens 5, 11, 16) are biochar treatment. Third row (pens 4, 8, 12) are peatmix treatment. Bottom row (pens 3, 6, 18) are microbial additive treatment.
