# Supplementary material for: Influence of litter substrates on production, egg quality, and welfare indicators in laying hens
Source: Poult Sci. 2025 Dec 5;105(1):106201. doi: 10.1016/j.psj.2025.106201 (PMC12771291; doi:10.1016/j.psj.2025.106201)
Supplement: Supplementary file 2 [file mmc2.docx]

Supplementary Materials

Supplementary table 1. Weekly replenishment options of litter materials on equal DM bases.

| Options | Wood Shavings ^1^ | Biochar + Wood shavings^2^ | Peatmix | Substrate^3^ |
| --- | --- | --- | --- | --- |
|  | (g) as is | (g) as is | (g) as is | (g) DM |
| 1 | 780 | 1000 + 0 | 1400 | 700 |
| 2 | 1560 | 1000 + 780 | 2800 | 1400 |
| 3 | 2340 | 1000 + 1560 | 4200 | 2100 |
| 4 | 3120 | 1000 + 2340 | 5600 | 2800 |

^1^ The quantity of wood shavings was the same for Wood shavings and microbial additive treatments.

^2^ Depending on the option, the quantity of wood shavings varied while the quantity of biochar was maintained at 700 g DM. A range of 30 -100% weekly dosage, which is higher than the dosages reported in literature (Gerlach & Schmidt 2012; Flores et al. 2021).

^3^ Explanation for treatment dosages:

- Peatmix- This is an already mixed commercial product in Sweden used by animal farmers as litter materials. It contains 80% peat and 20% wood shavings. We investigated 100% peat but it had very high moisture content.
- Biochar- Weekly quantity maintained at 700 g DM based on 1 g per hen per day. A calculation based on previous study in young bulls where biochar was added to a deep litter to mitigate greenhouse gas emissions (Nadeau et al. 2022). Assumption: A hen produces 30 g DM manure per day. If 10 g falls in the litter area, then 10 % amendment will be 1 g biochar per hen per day.
- Microbial additive- Dosage on label is 1-3kg /m3 for composting. But it was modified to 1g per hen per week as advised by the manufacturer.

Supplementary table 2. Effect of litter strategies on proportions of cracked and dirty eggs among laying hens. Values presented are least square means with their standard errors.

| Item | Cracked Egg (%) | Dirty Egg (%) |
| --- | --- | --- |
| Treatment^1^ |  |  |
| Wood Shavings | 0.93 | 25.0 |
| Peatmix | 0.80 | 30.5 |
| Biochar | 0.75 | 30.5 |
| Additive | 1.03 | 25.4 |
| SE | 0.15 | 1.77 |
| Hen age |  |  |
| 32 weeks | 0.36 ± 0.08^a^ | 35.2 ± 0.8^a^ |
| 38 weeks | 0.85 ± 0.14^b^ | 32.1 ± 2.3^a^ |
| 49 weeks | 1.42 ± 0.17^c^ | 16.3 ± 1.0^b^ |
| P-value |  |  |
| Treatment (T) | 0.481 | 0.002* |
| Hen Age (A) | <0.001 | <0.001 |
| T x A | 0.272 | 0.978 |
|  |  |  |

^1^ Wood shavings- treatment was pure wood shaving; Peatmix- treatment was a mixture of 80% peat and 20% wood shavings; Biochar- treatment was a combination of biochar and wood shavings; Additive- treatment was wood shavings with microbial additive.

^abc^ Means within a column of the section hen age lacking a common superscript are significantly different (P<0.05).

SE is Standard error of least square means.

*Difference in proportion of dirty eggs was not evident after the Tukey-Kramer adjustment for multiple comparisons.

Supplementary table 3. Effect of litter strategy on interior egg quality parameters of laying hens. Values presented are least square means with their standard errors

|  | Egg | Albumen | Haugh unit | Shell thickness | Breaking | Albumen |
| --- | --- | --- | --- | --- | --- | --- |
| Item | Weight (g) | height (mm) | (HU) | (mm) | strength (kg) | DM (%) |
| Treatment^1^ |  |  |  |  |  |  |
| Wood Shaving | 64.1 | 9.51 | 95.7 | 0.376 | 4.69 | 11.3 |
| Peatmix | 64.2 | 9.45 | 95.4 | 0.374 | 4.56 | 11.3 |
| Biochar | 63.8 | 9.37 | 95.3 | 0.377 | 4.67 | 11.2 |
| Additive | 63.7 | 9.52 | 96.0 | 0.372 | 4.64 | 11.4 |
| SE | 0.4 | 0.13 | 0.58 | 0.002 | 0.08 | 0.07 |
| Hen age |  |  |  |  |  |  |
| 39 wk | 63.9± 0.4^ab^ | 10.53± 0.11^a^ | 100.7± 0.48^a^ | 0.374± 0.002^a^ | 4.71± 0.07 | 11.5± 0.06^a^ |
| 43 wk | 65.0± 0.4^a^ | 9.17± 0.09^b^ | 94.1± 0.42^b^ | 0.380± 0.002^b^ | 4.65± 0.06 | 11.3± 0.05^b^ |
| 50 wk | 63.1± 0.3^b^ | 8.68± 0.08^c^ | 92.0± 0.40^c^ | 0.370± 0.002^a^ | 4.56± 0.05 | 11.1± 0.04^c^ |
| Day^2^ |  |  |  |  |  |  |
| 1 | 63.6 | 9.76^a^ | 97.1^a^ | 0.375 | 4.63 | 11.4 |
| 2 | 64.3 | 9.49^b^ | 95.7^b^ | 0.374 | 4.63 | 11.3 |
| 3 | 64.0 | 9.12^c^ | 94.0^c^ | 0.375 | 4.67 | 11.2 |
| SE | 0.3 | 0.09 | 0.43 | 0.002 | 0.06 | 0.05 |
| P-value |  |  |  |  |  |  |
| Treatment (T) | 0.589 | 0.910 | 0.843 | 0.484 | 0.651 | 0.546 |
| Hen age (A) | <0.001 | <0.001 | <0.001 | <0.001 | 0.188 | <0.001 |
| Day | 0.303 | <0.001 | <0.001 | 0.832 | 0.831 | 0.041 |
| T x A | 0.707 | 0.592 | 0.367 | 0.844 | 0.779 | 0.784 |

^1^Wood shavings- treatment was pure wood shavings; Peatmix- treatment was a mixture of 80% peat and 20% wood shavings; Biochar- treatment was a combination of biochar and wood shavings (Table 1); Additive- treatment was wood shavings with microbial additive.

^2^ Day is the number of days an egg spent in storage before measurements were taken.

^abc^ Means within a column of a section lacking a common superscript are significantly different (P<0.05).

SE is Standard error of least square means

Supplementary table 4. Effect of litter strategies on live body weight and integument score of laying hens. Values presented are least square means. Higher scores indicate better conditions.

| Item | Hen | Feather^2^ | Feather^3^ | Feet^3^ | Peck injury^3^ | | Bumble^3^ |
| --- | --- | --- | --- | --- | --- | --- | --- |
|  | Weight (g) | cover | cleanliness | cleanliness | Rear | Comb | foot |
| Treatment^1^ |  |  |  |  |  |  |  |
| Wood Shavings | 1742 | 19.1 | 2.59 | 3.33 | 3.45 | 2.77 | 3.95 |
| Peatmix | 1750 | 17.5 | 2.93 | 3.38 | 3.29 | 2.62 | 3.91 |
| Biochar | 1735 | 17.5 | 2.76 | 3.00 | 3.44 | 2.74 | 3.91 |
| Additive | 1755 | 17.6 | 2.77 | 3.31 | 3.48 | 2.78 | 3.88 |
| SE | 10.7 | 0.8 | 0.07 | 0.12 | 0.21 | 0.07 | 0.03 |
| Age |  |  |  |  |  |  |  |
| 36 wk | 1716^a^ | 21.1^a^ | 2.86 | 2.96^a^ | 3.74^a^ | 2.66^b^ | 3.91 |
| 50 wk | 1775^b^ | 14.7^b^ | 2.67 | 3.55^b^ | 3.09^b^ | 2.79^a^ | 3.92 |
| SE | 7.5 | 0.4 | 0.04 | 0.06 | 0.11 | 0.04 | 0.02 |
| P-value |  |  |  |  |  |  |  |
| Treatment (T) | 0.653 | 0.639 | 0.318 | 0.247 | 0.138 | 0.174 | 0.850 |
| Age (A) | <0.001 | <0.001 | 0.180 | 0.006 | 0.038 | 0.013 | 1.000 |
| T x A | 0.439 | <0.001* | 0.758 | 0.155 | 0.019* | 0.142 | 1.000 |

^1^Wood shavings- treatment was pure wood shavings; Peatmix- treatment was a mixture of 80% peat and 20% wood shavings; Biochar- treatment was a combination of biochar and wood shavings (Table 1); Additive- treatment was wood shavings with microbial additive.

^2^ Score for combined feather condition (rear, back, wings, neck, breast, and tail) from 6 to 24 where a higher score indicates a better feather cover.

^3^ Scores shown are mean values of untransformed data (not the binary outcome).

^abc^ Means within a column of a section lacking a common superscript are significantly different (P<0.05).

SE is Standard error of least square means.

*Difference was not evident after the Tukey-Kramer adjustment for multiple comparisons

Supplementary table 5. The number of hen and calculated available spaces per hen during behavior observation. Values are averages ± standard deviation.

| Treatment | Number of hens on perch | Number of hens in litter | Number of hens on^1^ slat | Slat space^1^ (cm^2^/ hen) | Litter space (cm^2^/ hen) |
| --- | --- | --- | --- | --- | --- |
| Biochar | 21.2 ± 5.7 | 16.7 ± 5.3 | 50.0 ± 7.3 | 1829.0 ± 294.8 | 3124.6 ± 1043.8 |
| M additive | 22.2 ± 6.2 | 17.3 ± 6.2 | 45.1. ± 6.9 | 1864.3 ± 306.8 | 3135.1 ± 1312.8 |
| Peatmix | 22.6 ± 6.4 | 19.5 ± 5.7 | 51.3 ± 6.5 | 1626.7 ± 219.3 | 2631.5 ± 797.8 |
| W Shavings | 20.1 ± 5.9 | 17.5 ± 5.6 | 49.6 ± 5.9 | 1675.9 ± 199.1 | 3001.8 ± 1122.4 |

^1^ Average available space per hen here, assumes that hen that were not in the litter or on the perch were on the slatted floor. Slatted area was 8.2 m^2^ and litter area was 4.7 m^2^

Supplementary table 6. Water holding capacity (WHC) and distribution of particle sizes substrate material at room temperature.

|  |  |  | Particles Size (%) | | | | |
| --- | --- | --- | --- | --- | --- | --- | --- |
|  | DM | WHC | < 0.4 | 0.4 -1.4 | 1.4 -2.5 | 2.5 - 4 | >4 |
| Material | (%) | (%) | mm | mm | mm | mm | mm |
| Wood shavings | 91.9 | 265.55 | 1.4 | 15.78 | 27.92 | 23.11 | 31.79 |
| Peatmix | 90.2 | 418.78 | 24.64 | 23.31 | 12.33 | 11.04 | 28.67 |
| Biochar | 83.8 | 234.94 | 12.6 | 32.55 | 20.95 | 15.07 | 18.82 |

References

Nadeau, E., Jeppsson, Knut-Håkan, Jansson, Anna, Wallin, Stefan, Hermansson, Cecilia, Hallin, Ola & Eliasson, K.A. (2022). Slutrapport till Jordbruksverket av projektet Biokol för minskat utsläpp av ammoniak och växthusgaser på nötköttsgård med biogas. Jordbruksverkets, 2017–4257. https://5dok.org/document/y960kl5v-slutrapport-jordbruksverket-projektet-biokol-utsl%C3%A4pp-ammoniak-v%C3%A4xthusgaser-n%C3%B6tk%C3%B6ttsg%C3%A5rd.html [2025-11-13]

Gerlach, H. & Schmidt, H.-P. (2012). Biochar in poultry farming. *Ithaka Journal*, 1/2012, 262–264

Flores, K.R., Fahrenholz, A. & Grimes, J.L. (2021). Effect of pellet quality and biochar litter amendment on male turkey performance. Poultry Science, 100 (4), 101002. https://doi.org/10.1016/j.psj.2021.01.025
